# Contribution of Amyloid Precursor Protein towards Mitochondrial Dysfunction in Alzheimer's Disease

**DOI:** 10.1002/alz70855_103247

**Published:** 2025-12-27

**Authors:** Kriti Shukla, Zhi Zhang, Casandra Salinas, Dominika Siodlak, Samah Houmam, Dylan Barber, Jialing Lin, Heather C. Rice

**Affiliations:** ^1^ University of Oklahoma, Norman, OK, USA; ^2^ University of Oklahoma Health Science Center, Oklahoma City, OK, USA; ^3^ Oklahoma Medical Research Foundation, Oklahoma City, OK, USA

## Abstract

**Background:**

Mitochondrial dysfunction is a key feature of Alzheimer's disease (AD). Amyloid Precursor Protein (APP), the precursor to Aβ peptides that form amyloid plaques in AD, has been shown to localize to the mitochondria under certain conditions. However, the precise role of APP in mitochondria is not fully understood, and exploring its protein‐protein interactions could provide crucial insights. Recent work by Rice et al. identified PGAM5, a mitochondrial phosphatase, as a candidate interactor of APP. PGAM5 is involved in regulating important mitochondrial processes such as fission, fusion, and mitophagy through interactions with various substrates. We sought to identify the role of APP in mitochondrial functions in health and AD through its interaction with PGAM5.

**Method:**

To characterize the binding between APP and PGAM5, we performed in vitro pull‐down assays and isothermal calorimetry (ITC). Proximity ligation assays in mouse brain tissue and primary astrocytes were employed to examine the endogenous interaction of these proteins. Subcellular fractionations were performed to study the processing and localization of APP and PGAM5 in wildtype and APP NL‐G‐F mice brains.

**Result:**

Pull‐down assays revealed that the linker region of APP binds to either the multimerization motif or the Keap‐1 binding domain of PGAM5. ITC demonstrated an interaction between APP and PGAM5. Endogenous interactions between APP and PGAM5 were detected in the brains of healthy mice and primary astrocytes, with potential location of this interaction being the mitochondria‐associated membranes (MAMs). Brain fractionation data demonstrated that both PGAM5 and full‐length APP (APP‐FL) were present in MAMs, while APP carboxy‐terminal fragments (APP‐CTF) localized to mitochondria along with PGAM5. Additionally, PGAM5 cleavage was found to be reduced in the brains of older AD mice.

**Conclusion:**

Our findings suggest that APP and PGAM5 interact under physiological conditions. In AD, the decreased cleavage of PGAM5 may impair mitophagy, contributing to the mitochondrial dysfunction seen in AD pathogenesis. Precise understanding of the interaction of APP with other proteins, like PGAM5 is critical to ensure the long‐term safety of potential treatments like APP knockdown in AD patients.

